# Experimental Approaches for Characterizing the Endocrine-Disrupting Effects of Environmental Chemicals in Fish

**DOI:** 10.3389/fendo.2020.619361

**Published:** 2021-02-25

**Authors:** Fritzie T. Celino-Brady, Darren T. Lerner, Andre P. Seale

**Affiliations:** ^1^ Department of Human Nutrition, Food and Animal Sciences, University of Hawai’i at Mānoa, Honolulu, HI, United States; ^2^ University of Hawai’i Sea Grant College Program, University of Hawai’i at Mānoa, Honolulu, HI, United States

**Keywords:** arsenic, estrogenic endocrine-disrupting chemicals, growth, *in silico*, methods, microplastics, plasticizers, reproduction

## Abstract

Increasing industrial and agricultural activities have led to a disturbing increase of pollutant discharges into the environment. Most of these pollutants can induce short-term, sustained or delayed impacts on developmental, physiological, and behavioral processes that are often regulated by the endocrine system in vertebrates, including fish, thus they are termed endocrine-disrupting chemicals (EDCs). Physiological impacts resulting from the exposure of these vertebrates to EDCs include abnormalities in growth and reproductive development, as many of the prevalent chemicals are capable of binding the receptors to sex steroid hormones. The approaches employed to investigate the action and impact of EDCs is largely dependent on the specific life history and habitat of each species, and the type of chemical that organisms are exposed to. Aquatic vertebrates, such as fish, are among the first organisms to be affected by waterborne EDCs, an attribute that has justified their wide-spread use as sentinel species. Many fish species are exposed to these chemicals in the wild, for either short or prolonged periods as larvae, adults, or both, thus, studies are typically designed to focus on either acute or chronic exposure at distinct developmental stages. The aim of this review is to provide an overview of the approaches and experimental methods commonly used to characterize the effects of some of the environmentally prevalent and emerging EDCs, including 17 α-ethinylestradiol, nonylphenol, BPA, phthalates, and arsenic; and the pervasive and potential carriers of EDCs, microplastics, on reproduction and growth. *In vivo* and *in vitro* studies are designed and employed to elucidate the direct effects of EDCs at the organismal and cellular levels, respectively. *In silico* approaches, on the other hand, comprise computational methods that have been more recently applied with the potential to replace extensive *in vitro* screening of EDCs. These approaches are discussed in light of model species, age and duration of EDC exposure.

## Introduction

For more than a decade, a growing body of evidence has demonstrated that anthropogenically introduced compounds alter functions of the vertebrate endocrine system (1, 2). These compounds have been termed “endocrine-disrupting chemicals” (EDCs). EDCs, as defined by the U.S. Environmental Protection Agency (EPA), are “exogenous compounds that interfere with the processing and action of endogenous hormones involved in the maintenance of homeostasis and regulation of development (3). With the knowledge obtained from recent studies, the extent of our understanding of how these chemicals exert their endocrine interfering effects have expanded. From a physiological point of view, EDCs are defined as natural or synthetic compounds that disrupt the hormonal and homeostatic systems that control how organisms communicate with and respond to the environment (4). Based on the consensus statement of La Merrill et al. (5), the key characteristics of EDCs include: interaction with or activation of hormone receptors; antagonistic effect on hormone receptors; alterations of hormone receptor expression, hormone synthesis, signal transduction in hormone-responsive cells, hormone transport across cell membranes, hormone distribution or circulating hormone levels, hormone metabolism or clearance, fate of hormone-producing or hormone-responsive cells; and induction of epigenetic modifications in hormone-producing or hormone-responsive cells. Although, only mechanistic evidence for a chemical can unequivocally classify it as an EDC, it is sometimes difficult to exclude adverse endocrine effects that are correlational in nature, especially from *in vivo* studies, and confounding non-specific effects of a chemical on the endocrine system that are not directly related to the mode of action of EDCs as described above (i.e., cell toxicity, tissue injury, and compensatory effects). Known EDCs include plastic additives, plasticizers, industrial solvents/lubricants and their byproducts, dioxins, alkyl phenols, pesticides, pharmaceuticals, drugs, anabolic agents, and naturally occurring compounds. These and additional examples of EDCs and their effects have been comprehensively reviewed elsewhere (6–9). The growing concern for EDCs has been focused on their effects on wildlife and humans (10, 11). Data from human studies and rodent models show that EDCs are implicated in male and female reproductive development disorders (12–14), breast cancer (15), prostate cancer (16, 17), and obesity (18–20). Together, these studies not only underscore the significant public health concern and costs associated with EDCs (21), but also justify further in-depth investigation.

In aquatic environments, fish have been a reliable indicator of endocrine disruption, as they are among the first animals exposed to waterborne pollutants. In contrast to stable sexual determination in mammals, the process of sexual determination and differentiation in fish can be influenced by environmental factors (22–26). The developmental plasticity of sexual determination in fish make them particularly vulnerable to environmental EDCs that pollute waters they inhabit. Because hormone receptors play a major role in the regulation of growth and reproduction, EDCs that bind these receptors (4) and exert agonistic or antagonistic actions can result in abnormal endocrine functions (27). In rodents and amphibians, dichlorodiphenyldichloroethylene (DDE)/dichlorodiphenyltrichloroethane (DDT), diethylstilbestrol (DES), and bisphenol A (BPA) bind both androgen and estrogen receptors (28–31). DDE, DDT, kepone, methoxychlor, nonylphenol (NP), and polychlorinated biphenyls (PCB), were found to have affinity to the seven-transmembrane receptor, G-protein-coupled estrogen receptor (GPR30), in human embryonic kidney 293 (HEK293) cell lines (32). In mammals and zebrafish, BPA, phthalates, DDT, polybrominated diphenyl ethers (PBDE), and PCB can bind and alter the expression of peroxisome proliferator-activated receptor (PPAR), a protein which plays a critical role in regulating metabolism, affecting lipid metabolism and reproduction (33–35). In fish, EDCs are known to affect fertility, sexual maturation, somatic growth, stress responses and induce cellular damage, largely through alterations in the levels of hormones and their receptors (36–42). Some EDCs are reported to bioaccumulate in fish (43, 44) and shown to be transferred to offspring through the transfer of lipids from yolk to embryo (45–47). The pathways through which EDCs can impact the physiology of organisms are not only a result of their ability to bind receptors (5). The high sensitivity of larval fish to EDCs due to their developing endocrine system (48, 49) underscores the need to specifically address the effects of early-life exposures to environmental chemicals through targeted approaches. Chemicals found in municipal and industrial wastewater discharges are found to be the most common EDCs of concern for aquatic life (50, 51). The natural and synthetic steroid estrogens, 17β-estradiol (E2), and 17 α-ethinylestradiol (EE2), and alkyl phenols such as nonylphenol (NP) are among the most pervasive EDCs found in the aquatic environment (52–56). These chemicals are commonly found in sewage effluent discharged into rivers (57) and affect fish at concentrations, that range from 4.5 to 25 ng/L and 0.84 to 200 µg/L, respectively (58–61). EE2, a primary estrogen component of most oral contraceptive pills, is discharged through municipal wastewater. Because EE2 cannot be effectively removed by the treatment process, it contaminates other clean water sources that mix with treated water (62, 63). Nonylphenol ethoxylates (NPEs), widely used as surfactants in industrial processes and products, are also discharged through domestic and industrial wastewater (64, 65). NP, a degradation product of NPE, persists in the environment and has been determined to reduce fecundity and fertility in Japanese medaka (*Oryzias latipes*) (66), lower plasma testosterone (T) in male carp (*Cyprinus carpio*) (67), reduce male to female ratios in wild tilapia (*Oreochromis niloticus*) (68), and decrease in gonadosomatic index (percentage of gonad weight per body weight, GSI) and developmental delay in courtship behavior in male guppy (*Poecilia reticulata*) (69). Heavy metals have also been found to contaminate the environment (70) and act as endocrine disruptors (71–73). In particular, arsenic pollution has posed serious risk to aquatic organisms due to its widespread presence in aquatic environments (74–77). Arsenic has been shown to disrupt the endocrine system in rodents, chicken, and fish (78–81). BPA and phthalates, two of the most popular chemical building blocks in the plastics industry, are ubiquitous environmental pollutants (82, 83). BPA has been found to leach from dental sealants (84), tin cans (85), and food packaging (86, 87). Phthalates can leach from food packaging (87), alcoholic beverages (88), PVC flooring (89), personal care products (90), and medicinal products (91, 92). Thus, these chemicals can be easily released into water bodies that ultimately affect aquatic animals. A number of studies suggest that these plasticizers induce developmental impairment *via* thyroid and growth hormone axes, and impact reproduction in mammals and aquatic species (93). More recently, microplastic (MP) contamination has become a growing global concern (94). MPs are defined as plastic that is less than 5 mm in diameter (95) resulting from the breakdown of larger plastics that are degraded (96), or from manufactured plastic microbeads present in scrubbing agents and personal care products. MPs have been reported to be ubiquitously present in open oceans (95, 97, 98), estuaries (99), beaches, surface waters, and marine sediment (95, 100–102). Although there is no evidence that MPs directly act as EDCs on organisms, indirect effects found may be linked to chemicals, including plasticizers, that are adsorbed or leached from MPs. Nonetheless, the heightened concern over the effects of MPs as carriers of EDCs in fish still warrants thorough investigation of this emerging class of environmental contamination. Reports on the ingestion of MPs by aquatic animals, including fish (103–106), further drive efforts to understand the physiological effects of harmful chemicals leaching from MPs. Despite the plethora of known EDCs, we focus this review on the six categories of chemicals above to underscore the diversity of approaches employed to characterize their effects in fish.

While substantial progress has been made toward the assessment of the effects of EDCs in fish, a number of experimental considerations provide distinct paths for interpreting the mechanisms of action and physiological consequences of exposure to these chemicals. For example, exposing an adult fish to EDCs may have very different consequences compared with exposing fish at an early-life stage or at a developmentally critical stage. Moreover, the effects of EDCs may not be limited to the exposed individual but also extend to its progenies. Hence, approaches have ranged from *in vivo* assessments of the impact of EDCs during early-life and adult stages, along with long-term and transgenerational effects, to *in vitro* assays designed to characterize the direct effects of putative EDCs along with their affinity to hormone receptors, to *in silico* predictive models that integrate an array of pathways sensitive to EDCs. Some studies that have employed these different approaches are listed in **Table 1** to provide a brief synopsis of the topics discussed in the following sections. Focusing on the disruption of growth and reproduction in fish, our aim here is to provide a brief overview of the approaches and experimental designs commonly employed to assess the effects of some of the pervasive and emerging EDCs, and to highlight the need of a strategy that considers combining the diverse approaches to study the adverse effects of EDCs on aquatic wildlife.

**Table 1:** List of studies used to analyze endocrine disrupting effects of NP, EE2, BPA, phthalates, arsenic, and microplastics with the corresponding approaches and endpoints.

Reference	Approach	Duration	Species	EDC	Endpoints
(107)	*In vivo*, sub-adult/adult, waterborne	15–30 days	*Colisa fasciatus*	Arsenic	Oocyte development, number and diameter of follicle nucleoli, follicular atresia
(108)	*In vivo*, sub-adult/adult, waterborne	15–30 days	*Colisa fasciatus*	Arsenic	Testicular development, Leydig cell diameter, testicular necrosis and pyknosis
(109)	*In vivo*, sub-adult/adult, waterborne	3 weeks	*Oncorhynchus mykiss*	EE2, NP	Plasma VTG, testicular growth
(110)	*In vivo*, early-life, i.p. injection	30 days	*Salmo salar*	NP	HSI, plasma VTG
(111)	*In vitro*, binding assay, cell/organ culture	48 h (hepatocyte culture)	*Oncorhynchus mykiss*	NP	ER affinity, hepatocyte *vtg*
(112)	*In vivo*, sub-adult/adult, early-life, waterborne	3–60 days	*Xiphophorus helleri*	NP, BPA	Growth, hepatic *vtg*, reproductive damage
(113)	*In vivo*, transgenerational, waterborne	164 days	*Pimephales promelas*	BPA	F0 body length and body weight, plasma VTG; F1 egg production and hatchability
(114)	*In vitro*, binding assay	48 h	*Oncorhynchus mykiss*	EE2, NP, sewage treatment effluent	Estrogenic activity
(115)	*In vivo*, early-life, waterborne	I year	*Oncorhynchus mykiss*	NP	Hepatic VTG, ZRP
(116)	*In vivo*, transgenerational, waterborne	3 months (10 days/month)	*Oncorhynchus mykiss*	NP	Hatching rate; intersex; plasma E2, T, and VTG
(117)	*In vivo*, sub-adult/adult, waterborne	3 weeks	*Oryzias latipes*	NP	Egg production, fertility, GSI, plasma VTG, spermatogenesis, testis-ova
(118)	*In vitro*, cell/organ culture, binding assay; *in vivo*, early-life, waterborne	3 days (cell/organ culture); eggs to sexually mature fish	*Oncorhynchus mykiss, Cyprinus carpio* (*in vitro*)*; Danio rerio* (*in vivo*)	EE2	VTG, ER
(119)	*In vivo*, early-life, waterborne	5 days	*Salmo salar*	NP	Body weight, plasma IGF1
(120)	*In vitro*, cell/organ culture	4 days	*Abramis brama*, *Cyprinus carpio*	EE2	VTG
(121)	*In vivo*, sub-adult/adult, waterborne	4 weeks	*Oreochromis mossambicus*	Arsenic	Growth rate
(122)	*In vitro*, binding assay; *in vivo*, sub-adult/adult, waterborne	3 weeks (*in vivo*)	*Danio rerio*	EE2, NP	Plasma VTG, GSI, estrogenicity
(123)	*In vitro*, cell/organ culture	15–30 days	*Anguilla japonica*	NP	Spermatogenesis progress, morphology of testicular cells
(124)	*In vivo*, sub-adult/adult, waterborne	24–168 h	*Danio rerio*	EE2	Plasma VTG, E2 and T; cell growth- related genes, hormone metabolism; steroid binding; sterol metabolism; lipid metabolism, hepatic *igf2* and *igfbp1*
(66)	*In vivo*, transgenerational, waterborne	21 days	*Oryzias latipes*	NP	Egg production, fertility, hatchability, hepatic VTG, time to hatching
(125)	*In vivo*, early-life, waterborne	3–6 days	*Oncorhynchus mykiss*	NP	Hepatic *vtg* and *erα*, brain *gnrh2*
(126)	*In vivo*, sub-adult/adult, waterborne	3 weeks	*Danio rerio*	EE2	Hepatic *vtg*, *er*, and *igfbp1*
(127)	*In vivo*, early-life, dietary	10–40 dpf	*Oreochromis niloticus*	EE2	Serum IGF-I, hepatic *igf1*, number of *igf1-*expressing hepatocyes, pituitary *gh*, sex ratio, body length, body weight
(128)	*In vivo*, sub-adult/adult, dietary	68 days	*Danio rerio*	Arsenic	Egg production, hepatic *vtg*, % hatch rate, number of spawns
(40)	*In vitro*, cell/organ culture	18 h, 6–15 days	*Anguilla japonica*	Arsenic	Germ cell proliferation, apoptosis, DNA damage, progesterone synthesis, 11-KT
(129)	*In vivo*, early-life, ovarian fluid	3 h	*Oncorhynchus mykiss*	BPA	Growth, hatching, yolk-absorption, whole embryo GH levels, *ghr, igfs*, and *igf* rs
(130)	*In vivo*, sub-adult/adult, waterborne	3 weeks	*Danio rerio*	Phthalate	GVBD, fecundity, ovulation; ovarian *lhr, mPRb, ptgs2*, BMP15*;* plasma VTG
(131)	*In vivo*, early-life, waterborne	14–91 days	*Poecilia reticulata*	Phthalate	Body weight, body length
(132)	*In vivo*, sub-adult/adult, waterborne	48 h	*Pimephales promelas*	EE2	Hepatic *erα* and *vtg*, testicular *ar* and *er*, testicular *cyp17*
(133)	*In vivo*, early-life, waterborne	embryo to swim-up stage	*Pimephales promelas*	EE2	Whole animal *lh* and *vtg*
(134)	*In vivo*, sub-adult/adult, waterborne	1–6 weeks	*Oryzias latipes*	EE2	Hepatic *vtg* and *chg*, oocyte marker 42Sp50, testis-ova, zona pellucida related-genes
(135)	*In vivo*, early-life, i.p. injection	10 days	*Salmo salar*	NP	Plasma IGF-I and GH
(136)	*In vivo*, sub-adult/adult, waterborne	1–6 weeks	*Oncorhynchus kisutch*	EE2	Gonadotropin and release-related contigs, hepatic *vtg*, pituitary *lhß*
(137)	*In vivo*, early-life, waterborne	96 h	*Pimephales promelas*	Phthalate	Whole animal E2 and T
(138)	*In vivo*, sub-adult/adult, dietary	60 days	*Micropterus salmoides*	EE2	Gonadal *cyp19a*, gonadal and hepatic *er*, plasma VTG, HSI, GSI
(139)	*In vivo*, early-life, waterborne	4 days	*Salmo salar*	EE2, NP	Hepatic and whole animal *vtg*, HSI, plasma VTG
(140)	*In vitro*, binding assay, cell/organ culture; *in vivo* early-life, waterborne	eggs -124 dpf (*in vivo*)	*Salmo trutta f. fario*	WWTPE	AR, ER, VTG
(141)	*In vitro*, binding assay	–	*Carassius auratus, Cyprinus carpio, Danio rerio, Gasterosteus aculeatus, Lepomis macrochirus*, *Oryzias latipes, Pimephales promelas, Poecilia* *Reticulata, Rutilus rutilus*	NP, BPA	ERα transactivation
(142)	*In vivo*, sub-adult/adult, dietary	2 months	*Oryzias latipes*	MPs	hepatic *chg, erα, vtg*
(60)	*In vivo*, transgenerational, waterborne	102 days	*Pimephales promelas*	EE2	offspring survival, juvenile production, reproductive capability
(143)	*In vivo*, transgenerational, waterborne	7 days	*Oryzias latipes*	BPA, EE2	F2 fertilization rate, F3 embryo survival
(144)	*In vivo*, early-life, ovarian fluid	3 h	*Oncorhynchus mykiss*	BPA	food conversion ratio, specific growth rate, whole animal *gh*, *ghr*, *igfs*, *igfr*
(145)	*In vitro*, cell/organ culture	48 h	*Oncorhynchus mykiss*	EE2	*er*, *vtg*, *ghr1*, *igfbp1*
(146)	*In vivo*, sub-adult/adult, waterborne	15–30 days	*Carassius auratus*	Phthalate	Sperm production, sperm motility and velocity, 11-KT, testicular *StAR*
(147)	*In silico*, molecular docking	–	*Oryzias latipes*	BPA, NP	architecture of ligand binding domains of ERs, formation of hydrogen bonds with ER1
(148)	*In vivo*, transgenerational, waterborne	6 months	*Danio rerio*	arsenic	offspring body mass
(149)	*In vivo*, sub-adult/adult, waterborne	7 days	*Oreochromis niloticus*	NP	anorexia
(150)	*In vivo*, sub-adult/adult, waterborne	15 days	*Danio rerio*	BPA	egg production, fertilization success, hepatic and gonadal genes involved in reproductive function and epigenetic processes, global DNA methylation
(151)	*In silico*, QSAR/structural alerts	–	–	estrogenic and androgenic chemicals	–
(152)	*In vivo*, early-life, waterborne	2–120 hpf	*Danio rerio*	BPA	hatching time, numbers of GNRH3 neurons, whole animal *kiss1, kiss1r*, *gnrh3*, *lh*, *fsh*, and *er*
(153)	*In vivo*, transgenerational, ovarian fluid	3 h	*Oncorhynchus mykiss*	BPA	F1 body mass, epigenetic modifications
(42)	*In vivo*, early-life, waterborne	4 days, 21 days	*Salmo salar*	EE2, NP	hepatic *vtg, erα*, *ghr*, *igf1* and *igf2, igfbps*; body mass, plasma GH and IGF1; total length
(154)	*In vivo*, early-life, waterborne	7 days	*Acanthochromis polyacanthus*	MPs	body mass
(155)	*In silico*, QSAR/structural alerts	–	–	chemicals affecting ovarian development	–
(156)	*In silico*, QSAR/structural alerts	–	–	EDCs affecting fish	prediction of toxicity and ER binding
(157)	*In vivo*, early-life, waterborne	21 days	*Oreochromis mossambicus*	NP	HSI, hepatic *erα, erβ*, *igf1, igfbp1b, igfbp2b*
(158)	*In silico*, molecular docking	–	*Danio rerio*	BPA, NP	interaction potential to ERα
(159)	*In vivo*, early-life, waterborne	2–4 weeks	*Platichthys stellatus*	arsenic	length and weight gain, condition factor
(160)	*In vivo*, transgenerational, waterborne	60 days	*Oryzias melastigma*	MPs	F0 gonadal maturation, plasma E2 and T, GSI, HSI, hepatic *vtg* and *chg*, gonadotropins, and steroid synthesis-related genes, F1 hatching rate and body length
(161)	*In vivo*, early-life, waterborne	30 days	*Carassius auratus*	BPA	brain *gnrh*, *fshβ* and *lhβ*, GSI, 11-KT, gonadal maturation, testicular *ar*
(162)	*In silico*, molecular docking; *in vivo*, sub-adult/adult, waterborne	14–28 days (*in vivo*)	*Goodea atripinnis*	BPA	ovary *foxl2*, interaction with residues in *foxl2*
(163)	*In vivo*, transgenerational, waterborne	8 hpf–21 dph	*Menidia beryllina*	EE2	F0 follicle atresia, sex ratio, *17β-hsd*, brain *cyp19b;* F1 hatching success, larval length, egg production; F2 larval survival rate, larval length; methylation of *17β-hsd* and *ar* across generations
(164)	*In vivo*, transgenerational, waterborne	8 hpf–21 dph	*Menidia beryllina*	EE2	biological processes and pathways representative of growth and reproduction; methylation of hormone receptors, steroidogenesis and sexual development- related genes across generations
(165)	*In vivo*, sub-adult/adult, waterborne	3 weeks	*Salmo trutta caspius*	NP	plasma E2, T and FSH
(166)	*In vivo*, early-life, waterborne	embryo- 120 hpf	*Danio rerio*	arsenic	whole animal GH, *ghr*, *igf2*, *igfbp3*, *igfbp2a*, *igfbp5b*

ar, androgen receptor; BMP15, bone morphogenetic protein-15; BPA, bisphenol A; chg, choriogenin; cyp17, cytochrome P-45017; cyp19b, cytochrome P450 aromatase; dpf, days post fertilization; dph, days post hatch; E2, 17β-estradiol; EE2, 17α-ethinylestradiol; Er, estrogen receptor; foxl2, forkhead box protein L2; fsh, follicle-stimulating hormone; gh, growth hormone; ghr, gh receptor; gnrh, gonadotropin-releasing hormone; GSI, gonadosomatic index; GVBD, germinal vesicle breakdown; hpf, hours post fertilization; 17β-hsd, 17β-hydroxysteroid dehydrogenases; HSI, hepatosomatic index; igf, insulin-like growth factor; igfr, igf receptor; igfbp, insulin-like growth factor binding proteins; 11-KT, 11-ketotestosterone; kiss1, kisspeptin1; kiss1r, kiss1 receptor; lh, luteinizing hormone; lhr, lh receptor; MPs, microplastics; mPRb, progesterone receptors; NP, nonylphenol; ptgs2, cyclooxygenase (COX)-2; StAR, steroidogenic acute regulatory protein; T, testosterone; vtg, vitellogenin; zrp, zona radiata protein.

## *In Vivo* Studies

Laboratory studies are used to confirm the correlational relationship between chemical pollutants in the environment and malformations or dysfunction observed in fish in the wild. The variety of approaches that are used reflects the diversity of fish species, their natural habitat, and the broad range of EDCs that they are exposed to. Each approach is most effective for addressing one particular question depending on the specific life history and habitat of each species, and the type of chemical fish are exposed to. These *in vivo* approaches employ a wide variety of experimental protocols, including exposure to chemicals presented in the water, those added to the diet, or compounds delivered through intraperitoneal injection. Exposure periods vary and concentrations of compounds used are either based on the levels found in the environment, those that elicit a physiological response or both. Fish models also differ among these studies, and can be generally subdivided by size into large and small fish species. Examples of the former include salmonids, tilapia, and bass, while the latter encompass zebrafish, medaka, and guppies. Larger fish models usually bear economic importance, include species of more relevance to aquaculture and conservation studies and can provide sufficient sampling of tissues for various types of analyses, but have longer life spans, require larger areas for rearing and trials and relatively higher maintenance costs. On the other hand, small fish models have a short life span, rapid development and reproductive rates, and low maintenance and husbandry costs, which provide versatility to their use as research models; their small size, however, limits the amount of various tissues and organs for analyses. Regardless of size class and experimental limitations, however, the rapid expansion in genomic approaches have allowed for the analysis of multiple fish species at various life-stages as models for investigating the effects of EDCs. This section will present various *in vivo* approaches for assessing the effects of EDCs on growth and reproduction taking into account differences between age, life stage, sex, exposure time and the fact that EDCs may affect not only the individual directly exposed to it, but also its progeny (4, 167–169). Among the commonly employed indicators for monitoring reproductive responses to EDC exposure in fish are GSI plasma sex steroid levels, steroidogenic enzymes, estrogen and androgen receptors (Er and Ar, respectively), and vitellogenin (VTG). VTG, an estrogen-dependent yolk protein precursor, is a common biomarker for exposure to estrogenic compounds. In normal conditions, VTG is present only in females, but males may express VTG in response to environmental estrogens and commercial diets containing estrogens (170). The actions of both natural steroid hormones and exogenous hormone mimics are mediated by steroid hormone receptors (171, 172). The synthesis of VTG is stimulated by activation of estrogen receptors (ERs) (173–176). Hence, VTG and ERs have been widely used in both *in vivo* and *in vitro* experimental designs to assess the effects of EDCs (172, 177–184). The growth hormone (GH)/insulin-like growth-factor (IGF) axis is the principal endocrine system regulating the growth and development of vertebrates, including teleosts (185, 186). GH stimulates the production of IGFs upon binding to its receptor, GHR; both GH and IGFs promote growth in target tissues (187–191). IGF binding proteins (IGFBPs), modulate the actions of IGFs by affecting their availability and activities (185, 192, 193). In addition to morphological indicators of growth such as length, weight and condition factor, the factors involved in the GH/IGF system can therefore be applied as molecular markers for growth and the assessment of the effects of EDCs on growth in fish. We divide this section into studies that examine the effects of EDCs on adult, subadult, larval stages, and across generations.

### Sub-Adult/Adult Exposure

For decades, studies on the effects of EDCs have been geared toward determining the relationship between environmental chemical levels and the unnatural changes that lead to morphological abnormalities observed in wild fish (194–198). Many experimental approaches have focused on characterizing the effects of EDCs on sub-adult and adult fish. The large body mass of sub-adult and adult fish allows for the collection and analysis of numerous markers on several different tissues. On the other hand, adult fish being equipped with fully differentiated tissues and a well-developed endocrine system can more readily compensate from the effects of exogenous agents at environmental concentrations, thereby limiting the number of observable EDC effects.

Many studies have subjected adult fish to prolonged periods of EDCs exposure, while others have focused on short-term or acute exposure protocols. Since there are many definitions of chronic and acute exposure depending on the life cycle of the organism being studied, in this review, we define periods of acute exposure to EDCs as 1 week or less and sub-chronic/chronic exposure as more than 1 week to several months or years, regardless of the life-stage. In both paradigms, exposure to EDCs typically occur through waterborne exposure, diet or intraperitoneal injection. Physiological responses of fish may vary with different routes of exposure since this could affect bioaccumulation and/or bioavailability of contaminants. Bioaccumulation through these different routes may differ since the co-determinants for bioaccumulation are the various elimination mechanisms. For instance, accumulation of a waterborne contaminant by a non-dietary route can be alleviated by direct equilibrium exchange of the contaminants at the gill epithelium. Thus, dietary exposure may be quantitatively different from aqueous exposure (199). This difference, however, would still be life-stage dependent.

#### Acute Exposure

Acute exposure experiments evaluate EDCs that can elicit effects within a short period of time. This approach also allows investigators to obtain results expeditiously, thereby reducing cost of maintenance. The drawback of this method, however, is that it may overlook effects that are only apparent after a long period of exposure. Moreover, acute exposure of EDCs might not be suitable for adults of many larger fish species as they tend to have greater tolerance to chemical exposure, especially at low doses. Studies employing this approach are discussed below.

##### Reproduction

Because impairments in sexual development were among the first physiological effects to be observed as a result of exposure to EDCs, many studies on fish have focused on reproductive endpoints.

Experimental approaches employing acute exposure have been effective at detecting disruptive effects of NP and EE2 in adult fish. In reproductively mature male fathead minnows (*Pimephales promelas*) exposed to EE2 for 48 h, hepatic *er alpha* (*erα*) and *vtg*, and testicular *ar* and *er* were upregulated, while the testicular steroidogenic enzyme cytochrome P-45017 (*cyp17*) was downregulated (132). Aqueous exposure of gravid female zebrafish to EE2 elevated plasma VTG, reduced plasma E2 and T after 24 or 48 h, and affected the expression of genes involved in hormone metabolism, steroid binding, sterol metabolism, and cell growth in the liver after 24 and 168 h (124). A 1-week exposure to EE2 of sub-adult coho salmon (*Oncorhynchus kisutch*), increased hepatic *vtg* and pituitary *luteinizing hormone* (*lh*) *β* subunit mRNA, and induced *gonadotropin-releasing hormone* (*gnrh*) receptor contig in females (136). Adult male Swordtail fish (*Xiphophorus helleri)* exposed to NP and BPA for 3 days induced hepatic *vtg* mRNA expression and reproductive damage (112).

##### Growth

Assessments of acute exposure effects of EDCs in adult fish focus mainly on reproduction. Not many of these studies have focused on growth, however. Nile tilapia (*Oreochromis niloticus*) adult exposed to NP for 7 days exhibited anorexia (149). In gravid female zebrafish, gene ontology (GO) analysis showed that aqueous treatment with EE2 either downregulated or upregulated genes involved in the regulation of growth. Specifically, gene expression of *igf2* decreased by 24 and 168 h, while *igfbp1* was increased by 24 h and either decreased or increased by 168 h depending on the doses of EE2 (124). Genes involved in processes related to metabolism and lipid metabolism were downregulated by 168 h (124).

Generally, these acute exposure approaches on growth have mainly employed molecular marker targets, as changes in morphology or phenotype usually manifest after long periods of exposure.

#### Sub-Chronic/Chronic Exposure

Long-term exposure experiments represent a powerful tool to test EDCs that affect animals after a prolonged period. This approach better reflects the actual exposure of animals in the environment compared with acute exposure experiments.

##### Reproduction

Similar to acute exposure experiments, a large number of studies characterize the sub-chronic and chronic effects of EDCs on reproduction in adult fish. For instance, waterborne exposure of fish to NP or EE2 for 3 weeks increased plasma VTG and suppressed testicular growth in adult male rainbow trout, (*Oncorhynchus mykiss)* (109). In adult male and female Japanese medaka (*Oryzias latipes*), exposure of up to 6 weeks to EE2 or NP induced testis-ova in males, hepatic *vtg* and *choriogenin* (*chg*), ovarian development-related genes in testis, reduction in GSI in males, and decreased egg production and fertility (117, 134). Similarly, in sub-adult coho salmon, a 6-week exposure to EE2 increased hepatic *vtg* and pituitary *lhß subunit* mRNA, decreased *follicle-stimulating hormone* (*fsh*) *β* subunit mRNA, and induced gonadotropin synthesis and release-related contigs in females (136). A 3-week exposure of male and female Caspian brown trout (*Salmo trutta caspius)* to water containing NP resulted in increased levels of plasma E2 and decreased T and FSH levels in both sexes (165). Zebrafish exposed to EE2 for 3 weeks induced hepatic *vtg* and *er* mRNA expression (126). In a 60-day dietary exposure study, EE2-fed female largemouth bass (*Micropterus salmoides*) exhibited decreased plasma VTG, hepatosomatic index (HSI) and GSI, and increased hepatic and gonadal *er*, and gonadal aromatase (*cyp19a*) transcripts (138). Induction of *forkhead box protein L2* (*foxl2*), a gene which play a role in ovarian differentiation and in maintenance of ovarian functions in mammals and fish (200–202), was observed after 14 and 28 days of exposure to BPA in females, and after 28 days in males of killifish (*Goodea atripinnis*) (162). Breeding groups of zebrafish treated with BPA for 15 days exhibited increased egg production, reduced fertilization success, alterations in levels of genes involved in reproductive function and epigenetic processes and reduction in global DNA methylation in both liver and gonad tissue (150). Sperm production, motility and velocity, 11-ketotestosterone (11-KT), and testicular *steroidogenic acute regulatory protein* (*StAR*) were reduced following a 15 or 30-day exposure to phthalate in mature male goldfish (*Carassius auratus*) (146). Female zebrafish exposed to phthalate for 3 weeks exhibited a decrease in ovulation, fecundity, and germinal vesicle breakdown; a decrease in ovarian *lhr*, *progesterone receptors* (*mPRb*), and *cyclooxygenase (COX)-2* (*ptgs2*); and an increase in plasma VTG and ovarian bone morphogenetic protein-15 (BMP15) protein (130). *In vivo* studies testing the chronic effects of arsenic have also been effective at revealing disruption of reproduction in fish. In banded gourami (*Colisa fasciatus*), exposure to arsenic for 15 or 30 days, revealed decreased development of oocytes, decreased numbers and diameter of nucleoli, and increased numbers of atretic follicles in females (107). In a similar study, males were observed to have degenerative changes in the testicular lobules, reduction in the diameter of Leydig cells, and necrosis and pyknosis in testis (108). Female zebrafish fed with polychaete worms laden with metals including arsenic for 68 days showed a decrease in hepatic *vtg* transcripts, cumulative egg production, number of spawns, number of eggs per spawn, and % hatch rate (128).

Since it has only been recently that the risks of MP pollution and their associated chemicals have raised concern, there have been very limited studies on their effects, particularly on reproduction. In one of these studies, feeding adult Japanese medaka with diets containing 10% by weight marine-treated polyethylene virgin pellets for 2 months downregulated hepatic *chg* gene expression in males and hepatic *vtg*, *chg* and *erα* gene expression in females (142). Although this study only shows the effects of plastic treated with marine water, it suggests that MPs at environmentally relevant concentrations may indirectly alter endocrine system function in adult. A detailed overview of the studies conducted on the various effects of MPs on aquatic organisms can be found elsewhere (203).

##### Growth

While most research has focused on reproduction as an endpoint of EDC’s chronic effects, a limited number of studies have examined growth effects of selected EDCs in fish. Only few studies have been conducted so far on the effects of prolonged exposure to NP and EE2 on growth of sub-adult or adult fish. Nevertheless, some studies clearly showed effects of these EDCs on biomarkers of growth. For instance, in adult zebrafish exposed to EE2 for 3 weeks, hepatic *igfbp1* mRNA was downregulated (126). In a chronic arsenic toxicity study in the euryhaline and warmwater fish, Mozambique tilapia (*Oreochromis mossambicus*), aqueous exposure to sodium arsenite for 4 weeks decreased the growth rate of males in a dose-dependent manner (121).

Overall, these chronic and sub-chronic *in vivo* exposure approaches are capable of detecting not only the changes in the expression of growth and reproductive genes and proteins, but also phenotypic alterations associated with the exposure of sub-adults and adults to EDCs.

### Early-Life Exposure

**Figure 1:**
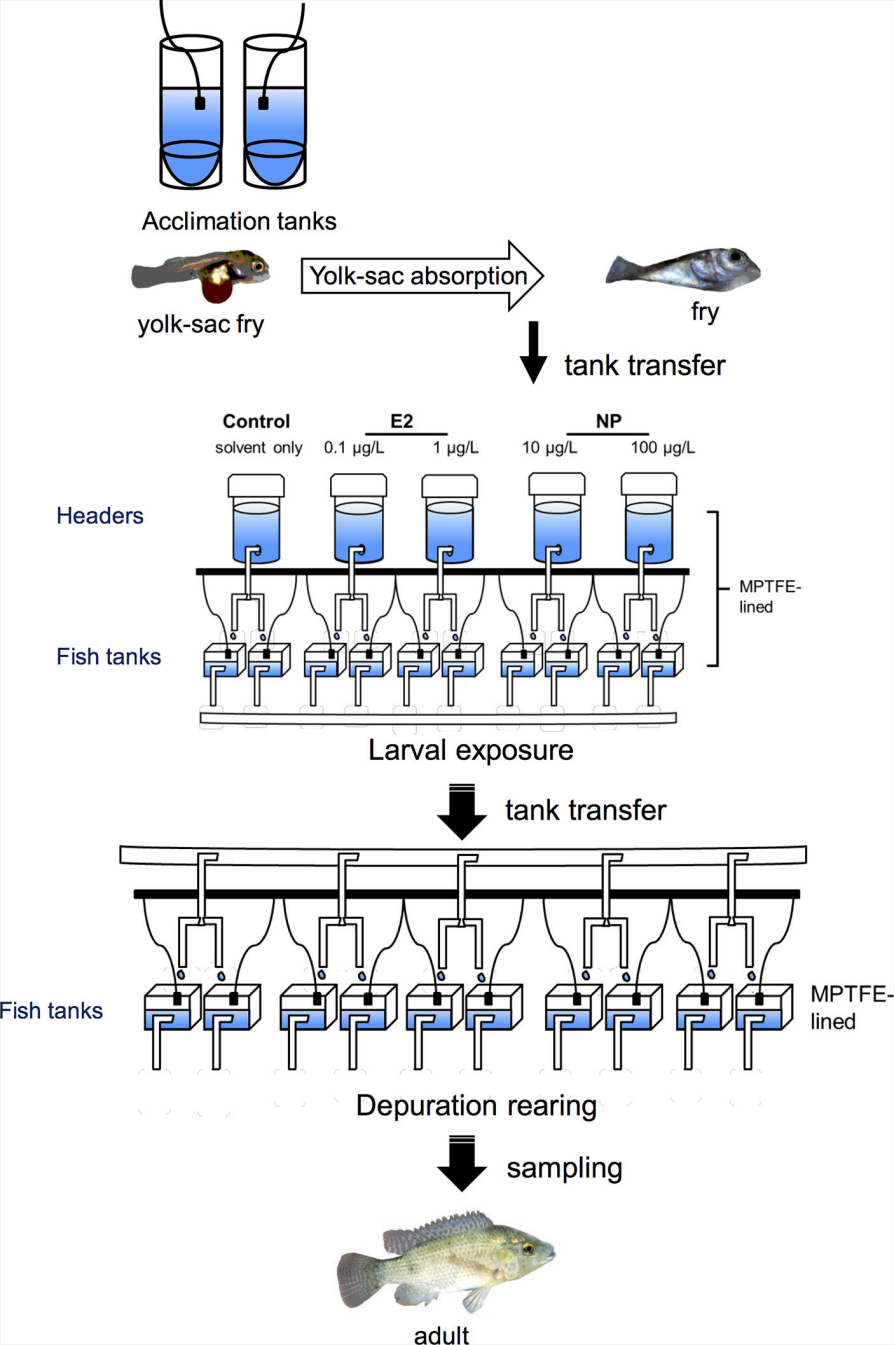
Schematic illustration depicting an experimental design of an *in vivo* exposure study aiming to assess the long-term developmental effects of early-life exposure to EDCs in fish [adapted from Celino-Brady et al. (157), with author’s permission]. This can be customized for a wide variety of species and EDCs. Yolk-sac fry are first allowed to acclimate to the experimental tanks and are then exposed to chemicals after partial or full yolk-sac resorption. Following the exposure to EDCs, fish are reared in EDC-free water (depuration) before sampling. In this experimental set up, both header and fish tanks are lined with modified polytetrafluoroethylene (MPTFE) to prevent the leaching of chemicals from the plastic containers. E2, 17β-estradiol; NP, nonylphenol. Black squares = aeration, white rectangles = pipes.

Examining the effects of EDCs during early life stages allows for the detection of abnormalities across the lifespan of fish, helping to clarify the sensitivity to specific chemicals during larval stages and long-term developmental effects. In some early-life stage experimental approaches, for example, the long-lasting effects of EDCs can be examined either right after exposure and/or after a period of depuration, even after fish have reached an adult stage. An example of an early-life, *in vivo* approach illustrating the methodology used for exposing Mozambique tilapia fry to NP and E2 and sampling after depuration is shown in **Figure 1**. Approaches employing larval fish also reduce the experimental footprint, or the effort and costs associated with the study, as they usually require smaller rearing and exposure containers (**Figure 1**). The lower the experimental footprint, the lower the effort and costs to conduct the study. In some studies, even petri dishes have been used for exposure experiments (133, 143). For acute exposures, however, particularly during very early stages the small size of the fish will restrict the amount and type of tissue samples and thereby limit the number and specificity of targets that can be used for analyses. It can also be difficult, if not impossible, to identify and separate sexes at early stages of development. In some cases, the challenge of having a limited amount of tissue was resolved by using the whole animal (133, 139, 166), although with this approach it is not possible to specify the organ or tissue from which gene expression or protein levels originate. In this case, methods such as *in situ hybridization* can be carried out in parallel to determine the expression patterns in different organs. If the whole animal is used, however, it is not possible to conduct this analysis in the same fish from which gene expression was determined. Moreover, determining the effects of separate sexes can be achieved by allowing the fish to grow to a stage by which sex can be identified. The high mortality rates during early developmental stages in fishes (204) also present a further challenge to this experimental strategy.

#### Acute Exposure

Acute exposures in early-life stages like in adult stages can cut down experimental footprints. Conducting this approach during very early stages, however, would make the size of the fish as a drawback limiting the number and specificity of biomarkers that can be analyzed. Below are some of the representative studies that used this type of exposure method.

##### Reproduction

Relatively short-exposure periods (days) were conducted in salmonid species. After 3 or 6 days of exposure to NP, hepatic *vtg* and *erα* mRNA levels were increased, while brain *gnrh2* mRNA were reduced in a dose-dependent manner in juvenile rainbow trout (125). Moreover, exposure to EE2 and NP for 4 days, elevated *vtg* transcripts in yolk-sac larvae, fry and smolts, and increased plasma VTG and *erα* mRNA levels in Atlantic salmon (*Salmo salar*) smolts (42, 139). An interesting experiment in which rainbow trout oocytes were exposed to BPA for 3 h in ovarian fluid so as to mimic maternal transfer, resulted in delay in hatching and yolk-absorption in embryo (129). Low levels of BPA exposure at 2 h post fertilization (hpf) to 120 hpf caused advanced hatching time, increased numbers of GNRH3 neurons and increased expression of reproduction-related genes such as *kisspeptin1 (kiss1) kiss receptor*, (*kiss1r)*, *gnrh3*, *lh*, *fsh*, and *er* in zebrafish larvae (152). In fathead minnow, sex steroid hormones (E2 and T) either increased or decreased after 96 h exposure of eggs to different types of phthalates (137).

##### Growth

Acute exposure of Atlantic salmon smolts to EE2 or NP for 4 days also impacted growth. EE2 diminished plasma GH and IGF1 levels in parallel with reductions in hepatic *ghr* and *igf1* (42). Smolt weights and plasma IGF-I levels were reduced by NP by 5 days of exposure (119). Rainbow trout embryo exposed to BPA for 3 h as oocytes exhibited growth impairment, increase in GH levels, and lower gene expression *ghr, igfs* and *igf* receptors (*igfrs)* (129). In a similar experiment, a decrease in the specific growth rate and food conversion ratio in rainbow trout larvae reared from BPA-treated eggs were observed. BPA also disrupted the mRNA levels of *gh* isoforms, *ghr*, *igfs*, *igfr* in a life stage-dependent manner (144). Zebrafish embryos exposed up to 120 hpf to arsenic had elevated GH levels, and reduced whole body *ghr, igf2, igfbp3, igfbp2a*, and *igfbp5b* (166). Although there is insufficient knowledge on the effects of MPs on growth, a one-week acute exposure study showed that growth was negatively affected by MPs in juvenile spiny chromis damselfish (*Acanthochromis polyacanthus)* (154). Nonetheless, the reduction in growth as a consequence of a reduction in feeding cannot be explicitly ruled out in this study. More studies designed to elucidate the direct effects and modes of action of MPs on growth are needed.

#### Sub-Chronic/Chronic Exposure

Chronic exposure approaches, although an effective tool for assessment of EDCs with delayed effects, have their own disadvantages if conducted during early stages of development. For example, prolonged exposure to chemicals can magnify the high mortality rates that are already a natural challenge during early-life development in fishes.

##### Reproduction

There have been more studies performed on the chronic early-life exposure effects of EDCs on the reproduction of fishes when compared with investigations of acute early-life effects. Long-term exposure (1 year) of rainbow trout to NP, from eyed-egg to the juvenile stage, resulted in the elevation of hepatic VTG expression and zona radiata protein (ZRP) (115). In a similar approach, fathead minnow embryos were continuously exposed to EE2 until swim-up stage, resulting in an increase in *lh* and *vtg* (133). Immature mixed sex Atlantic salmon injected with E2 or NP had higher HSI and elevated levels of plasma VTG by 30 days (110). In the same species, juvenile exposure to waterborne NP for 21 days induced plasma VTG in both males and females (36). Exposure of Atlantic salmon fry to EE2 or NP for 21 days resulted in increased hepatic *vtg* mRNA levels and decreased hepatic *erα* mRNA levels (42). In Mozambique tilapia, exposure of yolk-sac fry to waterborne NP for 21 days also resulted in alterations in reproductive-related genes. Specifically, a decrease in HSI, and stimulation of hepatic *erα* and *erβ* were observed in adult male fish 112 days after the end of the early-life exposure period (157). In Nile tilapia fry fed diets containing EE2 from 10–40 days post fertilization (dpf), sex ratio become skewed towards females (127). Three-month old goldfish subjected to aqueous exposure to BPA for 30 days demonstrated diminished ovarian maturation, decreased brain *gnrh*, *fshβ* and *lhβ* in females, and reduced GSI and testicular *ar* levels in male. Moreover, a decrease in 11-KT levels were also observed in BPA-exposed fish. The disruption of testicular development was not recovered after BPA withdrawal (161).

##### Growth

When fish are subjected to long-duration treatments with EDCs, their growth or growth-related physiological markers are also altered. Dietary exposure of Nile tilapia fry to EE2 from 10–40 dpf decreased serum IGF-I, hepatic *igf1* mRNA, the number of hepatocytes expressing *igf1*, and pituitary *gh* by 75 dpf. Both body length and weight also decreased after 90 to 165 dpf (127). In Atlantic salmon, fry subjected to waterborne EE2 or NP exposure for 21 days had decreased hepatic *ghr*, *igf1* and *igf2* mRNA levels, reduced body mass and total length, and varying effects on various *igfbp* transcript levels (42). Intraperitoneal injection of NP or E2 within a 10-day period in Atlantic salmon smolts decreased both plasma IGF-I and GH levels (125). Following a 21-day exposure of Mozambique tilapia yolk-sac fry to waterborne NP, stimulation of hepatic *igf1, igfbp1b*, and *igfbp2b* in adult male fish exposed as yolk-sac fry was observed (157). Sixty-day exposure of juvenile swordtail fish to both NP and BPA significantly reduced growth (112). Continuous exposure of less than one-week-old guppy (*Poecilia reticulata*) to phthalate for 14–91 days reduced body weight and body length (131). In juvenile starry flounder (*Platichthys stellatus*), daily length and weight gain, and condition factor decreased following 2 and 4 weeks of aqueous exposure to arsenic (159).

While these studies subjected fish to relatively long-term exposures (several weeks), the main difference between experimental designs is that most of the studies analyzed the effects of EDCs immediately following exposure (36, 42, 115, 133), while some assessed the effects of EDCs after a period of depuration in which fish were no longer fed with EDC-containing feed or moved to EDC-free water (127, 157). Notwithstanding, all of these studies showed significant changes in biomarkers of growth and reproduction following exposure to EDCs. In summary, studies employing both acute and chronic exposure protocols on fish at early-life stages were effective at detecting various impacts of EDCs on growth and reproduction.

### Transgenerational Studies

A number of studies in fish provide evidence of the disruptive effects of EDCs that span across parental and filial generations. Evidence from recent studies suggests that exposure to EDCs can directly impact not only the exposed individual, but also unexposed future progenies. This process is called transgenerational inheritance (167, 169). Transgenerational experiments only involve exposure of the parental or F0 generation to EDCs, with no exposure of subsequent generations. This can provide information on both direct and indirect consequences of EDC exposure. Furthermore, transgenerational effects are not limited to epigenetic changes such as DNA methylation, histone modification, and expression of non-coding RNAs (205) but also include the effects of the direct transfer of compounds *via* egg yolk and oocyte reserves. The main outcomes of determining the effects of parental exposure to EDCs on offspring have been highly variable and not strictly limited to endpoints of growth and reproduction. Our discussion focuses on the growth and reproduction-related biological markers and epigenetic changes. Moreover, only few studies employing a transgenerational approach have been conducted on the EDCs included in this review, hence this section is divided into acute and sub-chronic/chronic exposure.

#### Acute Exposure

Acute exposures to EDCs have been shown to affect filial generations in transgenerational studies. In Japanese medaka, aqueous EE2 and BPA treatments of late blastula stage for 7 days resulted in reduced fertilization rates in the F2 generation, and decreased embryo survival in F3 offspring (143). *In vivo* treatment of rainbow trout F0 oocyte with BPA for 3 h caused reduction in body mass in F1 generation. The long-term changes seen in the liver transcriptome of progenies raised from BPA-exposed eggs suggest epigenetic modifications (153).

#### Sub-Chronic/Chronic Exposure

In rainbow trout, male and female adults exposed to NP for 3 months induced an increase in plasma VTG in parental (F0) males, reduced hatching rates of eggs from F0 parents and elevated intersex frequency, and hormonal imbalance in male and female offspring (116). Exposure of adult Japanese medaka male and female pairs to NP for 21 days resulted in reduced egg production and fertility, elevated hepatic VTG levels in F0 males, and reduced hatchability and time to hatching of the F1 generation (66). In fathead minnow, rearing adult males and females in tanks with EE2 for 102 days caused reductions in F0 male survival and F1 juvenile production, reproductive failure or reduced reproductive capability in F1 adults and reduction in survival rates of F2 offspring (60). In inland silversides (*Menidia beryllina*), exposure to low levels of EE2 from 8 hpf to 21 days post hatch (dph), led to a female-biased sex ratio, higher number of atretic follicles, reduced *17β-hydroxysteroid dehydrogenases* (*17β-hsd*), and increased brain *cytochrome P450 aromatase* (*cyp19b)* in the F0 generation. In the F1 generation, hatching success increased, while larval length, and egg production decreased. Larval survival rate was reduced and larval length increased in the F2 generation. Moreover, differential methylation of *17β-hsd* and *ar* was found across generations (163). A similar study on the same species exposed to EE2 up to 21 dph, revealed changes in biological processes and pathways involved in growth, development and reproduction as assessed by GO and pathway analyses, and methylation of hormone receptors, steroidogenesis, and sexual development-related genes across generations (164). In fathead minnow, BPA inhibited body length and body weight in F0 males after up to 164 days of exposure. Plasma VTG levels in F0 parents were also elevated after 164 days of exposure. Egg production and hatchability were inhibited in the F1 generation after 164-days exposure (113). Furthermore, zebrafish chronically subjected to waterborne arsenic for 6 months had progeny whose body mass was lower than those from untreated parents (148). Marine medaka (*Oryzias melastigma*) exposed for 60-days to MPs showed a number of deleterious effecs including: 1) retardation of gonadal maturation, reduced plasma E2 and T, and decreased GSI of F0 female fish; 2) decrease in HSI and GSI in F0 males; 3) varying effects on gonadotropins and steroid synthesis-related genes in F0 parents; 4) increase in hepatic *vtg* and *chg* in F0 males; and 5) decrease in hatching rate and body length in progeny (160). As seen with other aforementioned MP studies, these effects may only be possible due to the leaching of plasticizers or to non-endocrine disrupting mechanisms, such as cell-toxicity, tissue-injury, or feeding inhibition.

The results from these transgenerational experiments show that acute EDC exposures are enough to induce transgenerational effects when parents are exposed at early stages. On the other hand, experiments carried out during adult stages may require longer exposure periods (weeks or months) to reveal transgenerational changes. Remarkably, these studies show that both F1 fish exposed to EDCs indirectly as primordial germ cells within F0 parents, and F2 animals not directly exposed to EDCs, exhibit physiological alterations induced by the parental EDC exposure.

## *In Vitro *Studies

**Figure 2:**
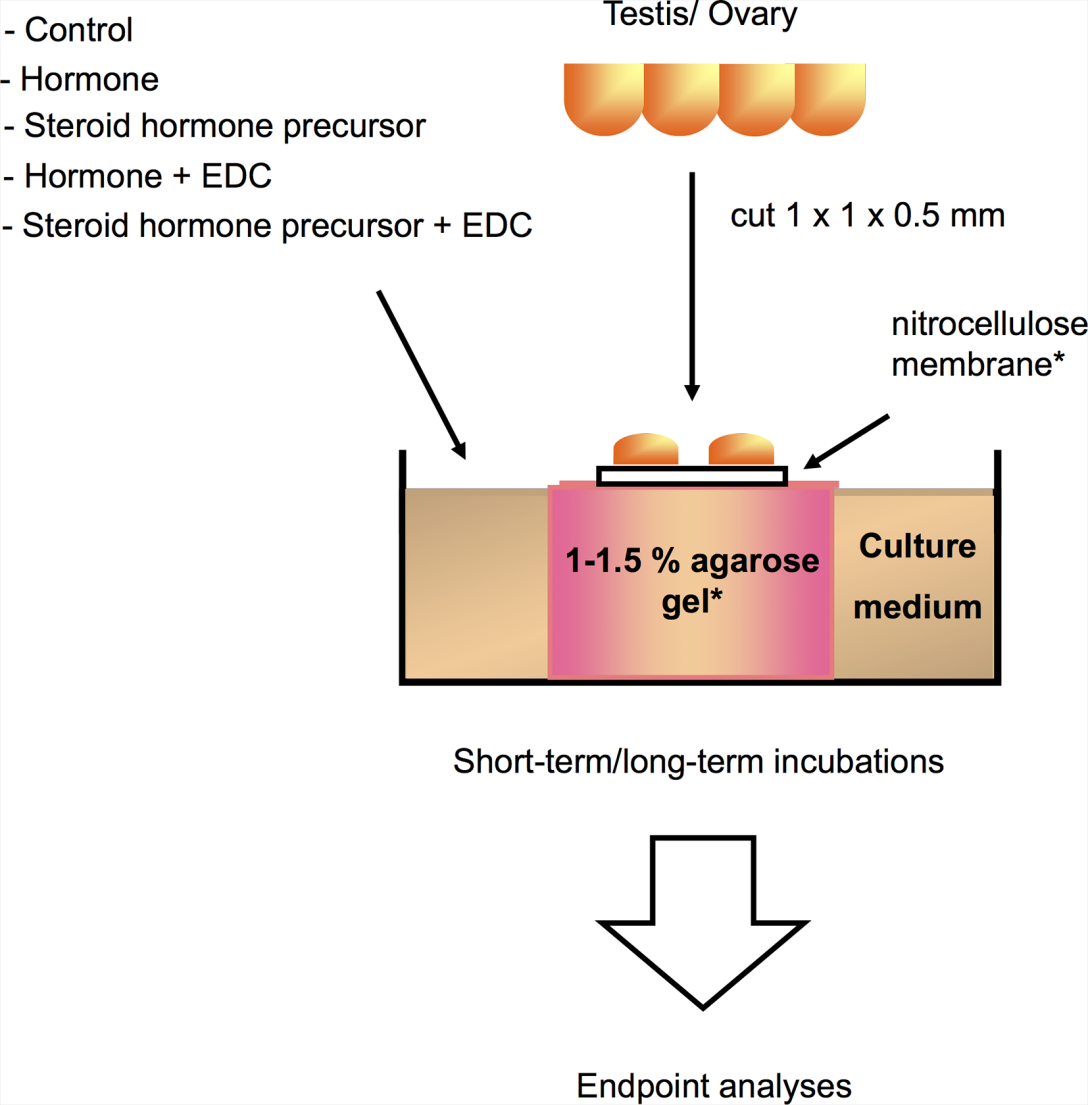
Schematic illustration of a gonadal *in vitro* culture system that can be utilized for analyzing direct effects of a variety of compounds or hormones on germ cell growth and development, as well as gonadal gene expression in different teleost species [illustration modified from Miura and Miura (206), with author’s permission]. Testis or ovary are first cut into small fragments, and then placed on a nitrocellulose membrane set on top of agarose gel cylinders for long-term incubations, or directly placed in the wells for short-term incubations. Testicular or ovarian fragments are cultured in medium with or without EDCs, hormone, or steroid hormone precursors for up to 30 days. Endpoint analyses correspond to immunoassays, histology, immunohistochemistry, and gene expression assays. *Usually utilized for long culture periods.

**Figure 3:**
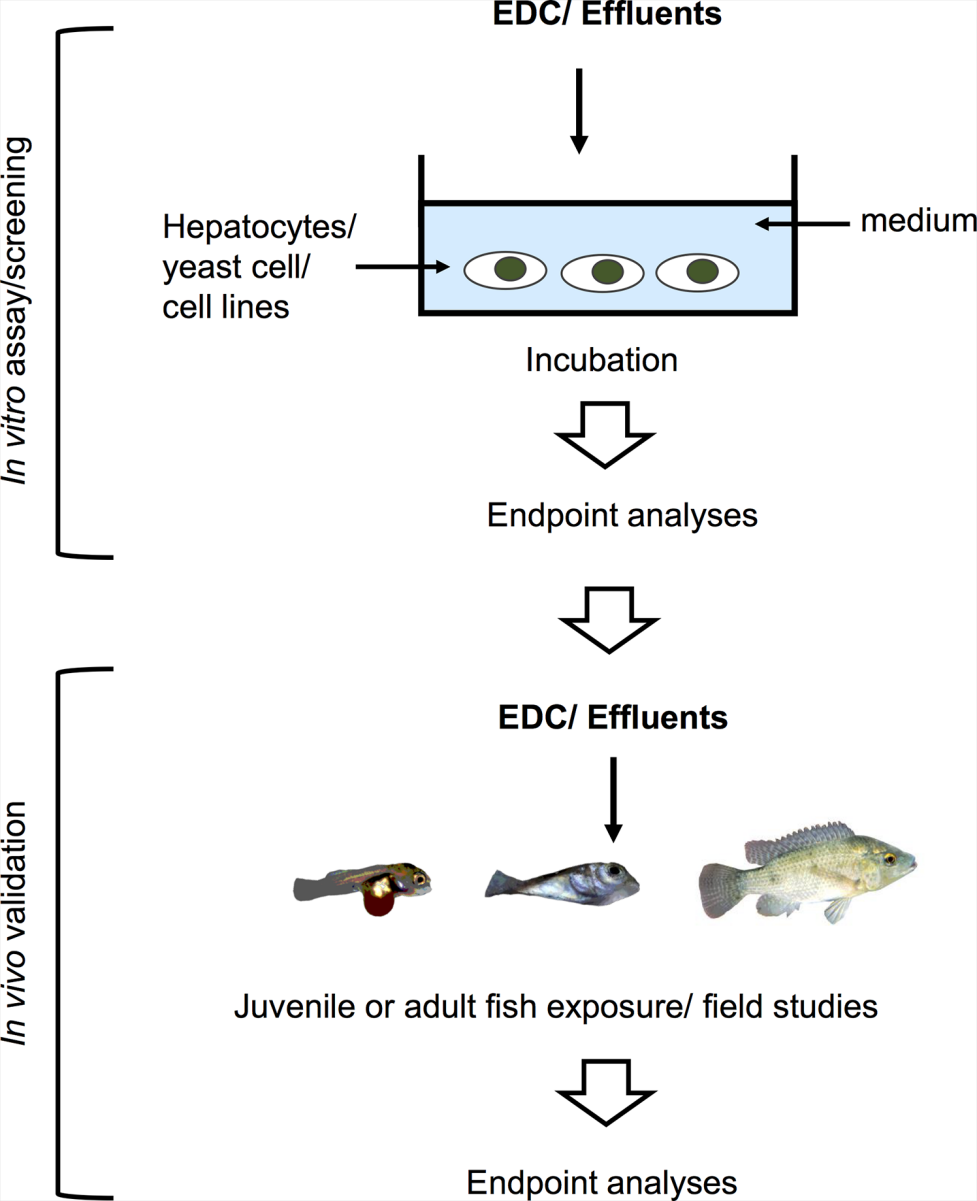
Schematic illustration of an *in vitro* system used for screening of potential EDCs and testing for estrogenicity of waste water effluents in a tiered strategy. These screening assays utilize fish and human cell lines, yeast cells, and human breast cancer cells for the first tier. The results are then validated in the lab by *in vivo* testing, or further validated by examining phenotypic or genotypic changes in fish inhabiting contaminated areas.

While *in vivo* experimental approaches take into account all physiological processes and interactions occurring in the animal upon EDCs exposure, *in vitro* approaches, on the other hand, are helpful in determining the direct effects of chemicals on a specific tissue or cell type. Since *in vitro* studies are inherently more controlled and include fewer confounding factors, they are suitable to determine specific mechanisms of action of EDCs under near-ideal conditions. Experimental manipulation of cellular pathways such as addition of specific inhibitors or activators, can help pinpoint the specific molecular target/s of EDCs within the cell, a key step in categorizing an EDC. These *in vitro* culture systems (exemplified in **Figure 2**) can address the effects of a wide variety of EDCs on cells or organs when coupled with methods to measure morphological changes, disruption on cell development and hormone synthesis related to growth and reproduction. In contrast to *in vivo* approaches*, in vitro* experimental designs are more temporally constrained as most cell or organ incubations have limited viability. As such, exposure time varies in *in vitro* systems based on viability of cells and the amount of time required to induce an effect on specific markers or responsive elements. An overview of several *in vitro* assays for substances possessing endocrine activity in fish is also provided by Scholz et al. (207). Some *in vitro* approaches are routinely used for screening candidate EDCs and wastewater samples (208–216), and used as a guide for a higher tier EDC test (*in vivo* experiments and field studies) in a tiered strategy (**Figure 3**). These *in vitro* screening assays include cell proliferation assays (E-screen assays), yeast-based screens and reporter gene assays that utilize fish and human cell lines, including human breast cancer cells. Further information on this tiered approach is discussed elsewhere (217, 218). We divide this section into cell/organ culture experiments and binding assays. Below, *in vitro* studies designed to analyze the direct effects of EDCs on hormone synthesis, ER/er, VTG/vtg, and GH/IGF-related factors, and to validate the endocrine disrupting potency of complex environmental chemical mixtures are discussed.

### Cell/Organ Culture

A short incubation (18 h) of testicular fragments with arsenic, steroid hormone precursors, and human chorionic gonadotropin (hCG) suppressed hCG-induced synthesis of 11-KT and inhibited progesterone synthesis from pregnenolone (40). Bream (*Abramis brama*) and carp hepatocytes treated with EE2 for 4 days showed concentration-dependent induction of VTG (220). Primary hepatocyte cultures have also been employed to characterize the effects of EE2 on growth-related factors. In rainbow trout juveniles, for example, hepatocytes treated with EE2 for 48 h showed a concentration-dependent upregulation of *er* and *vtg* and downregulation of *ghr1* and *igfbp1* (145). Treatment of testicular fragments of Japanese eel (*Anguilla japonica*) with NP with or without 11-KT for 15 or 30 days stimulated early spermatogonial renewal, induced hypertrophy in Sertoli cells and decreased the number of germ cells (123). Using the same *in vitro* system, testicular fragments cultured with arsenic in the presence or absence of hCG for 6 or 15 days elicited a dose-dependent inhibition of hCG-induced germ cell proliferation and induced apoptosis and oxidative DNA damage in germ cells (40). Some studies have coupled *in vitro* cell screening assays with *in vivo* approaches to provide a more comprehensive understanding of the effects of EDCs or to validate EDC potencies of wastewater treatment plant effluents (WWTPE) in fish. E-screen assays which is based on proliferation of human breast cancer cells MCF-7 is used to analyze the estrogenicity of water, sediment and WWTPE. This *in vitro* assay supported the results of *in vivo* experiments using juvenile brown trout (*Salmo trutta f. fario*) (140). Rainbow trout and carp hepatocyte assays were used to examine induction of VTG by EE2 and other compounds to predict their estrogenicity supporting the *in vivo* life cycle tests using zebrafish (118).

### Binding Assays

An *in vitro* screening assay using a recombinant yeast system expressing rainbow trout ER and a 48 h trout hepatocyte culture showed ER affinity and induction of hepatocyte *vtg* by NP, respectively (111). In another *in vitro* assay, estrogenic environmental chemicals were screened using the rainbow trout gonad cell line, RTG-2, containing *erα* complementary DNA (*rtErα* cDNA). Results indicated that EE2, NP and sewage treatment effluent extract had estrogenic activity (114). Other *in vitro* screening assays for assessing estrogenic activity in sewage treatment effluents are described in Rutishauser et al. (219). Miyagawa et al. (141) developed customized *in vitro* ERα reporter gene assays using HEK293 cells as hosts for ERα of various fish species [medaka, common carp, goldfish guppy, stickleback (*Gasterosteus aculeatus)*, bluegill (*Lepomis macrochirus*), fathead minnow, roach (*Rutilus rutilus*), and zebrafish] to analyze the ligand- and species-specificity for several estrogenic chemicals, including NP and BPA. NP and BPA induced the transactivation of ERα from all species of fish tested.

Several binding assays were also used in combination with *in vivo* experiments in a tiered approach. For instance, in zebrafish, *in vitro* screening assays using transfected human breast cancer cells (MVLN) containing an estrogen response element coupled to a luciferase reporter gene, and an estrogen-inducible expression system in yeast cells integrated with human estrogen receptor (YES screen) were used for dose-range and estrogenic potency studies to determine concentrations to be used in *in vivo* assays. EE2 and NP were found to be estrogenic in both *in vitro* assays and were able to induce plasma VTG and reduce the GSI in female zebrafish, *in vivo* (122). In another experiment, *in vitro* luciferase reporter assays employing human cell lines (HeLa-9903 and MDA-kb2) to detect ER or AR were used to analyze the endocrine activity of water, sediment, and WWTPE. The reproduction-disrupting effects revealed by these *in vitro* assays were subsequently corroborated by *in vivo* experiments using juvenile brown trout, thereby underscoring the power of high throughput *in vitro* approaches to narrow down the targets for endocrine disruption observed in field-exposed fish (140). In Segner et al. (118), the estrogenicity of compounds, including EE2, was tested by recombinant yeast ER assay, a carp hepatic ER competitive radioreceptor assay, and *in vivo* life cycle tests using zebrafish. In this study, EE2 estrogenicity was confirmed and the *in vitro* assay was found to be the best predictor of the *in vivo* test.

Unlike most *in vivo* approaches, these *in vitro* assays are usually rapid, cost-effective for the output of chemicals screened and can be performed under controlled environments, thereby eliminating the possibility of contamination by chemicals other than selected compounds to be tested. Some studies incorporate mammalian cell lines and luciferase reporter assays into *in vitro* approaches to evaluate dose responses of potential EDCs that can be used to test in fish models. Most of these *in vitro* tests have been designed to detect reproduction-related genes. For detecting growth-related genes, *in vitro* assays employing primary hepatocyte cultures have served as a good model for assessing the effects of EDCs on the GH/IGF system in fish (145). A combination of these *in vitro* tests provides a comprehensive perspective of the impact of EDCs on the growth and reproductive physiology of fish.

## *In Silico* Approaches

**Figure 4:**
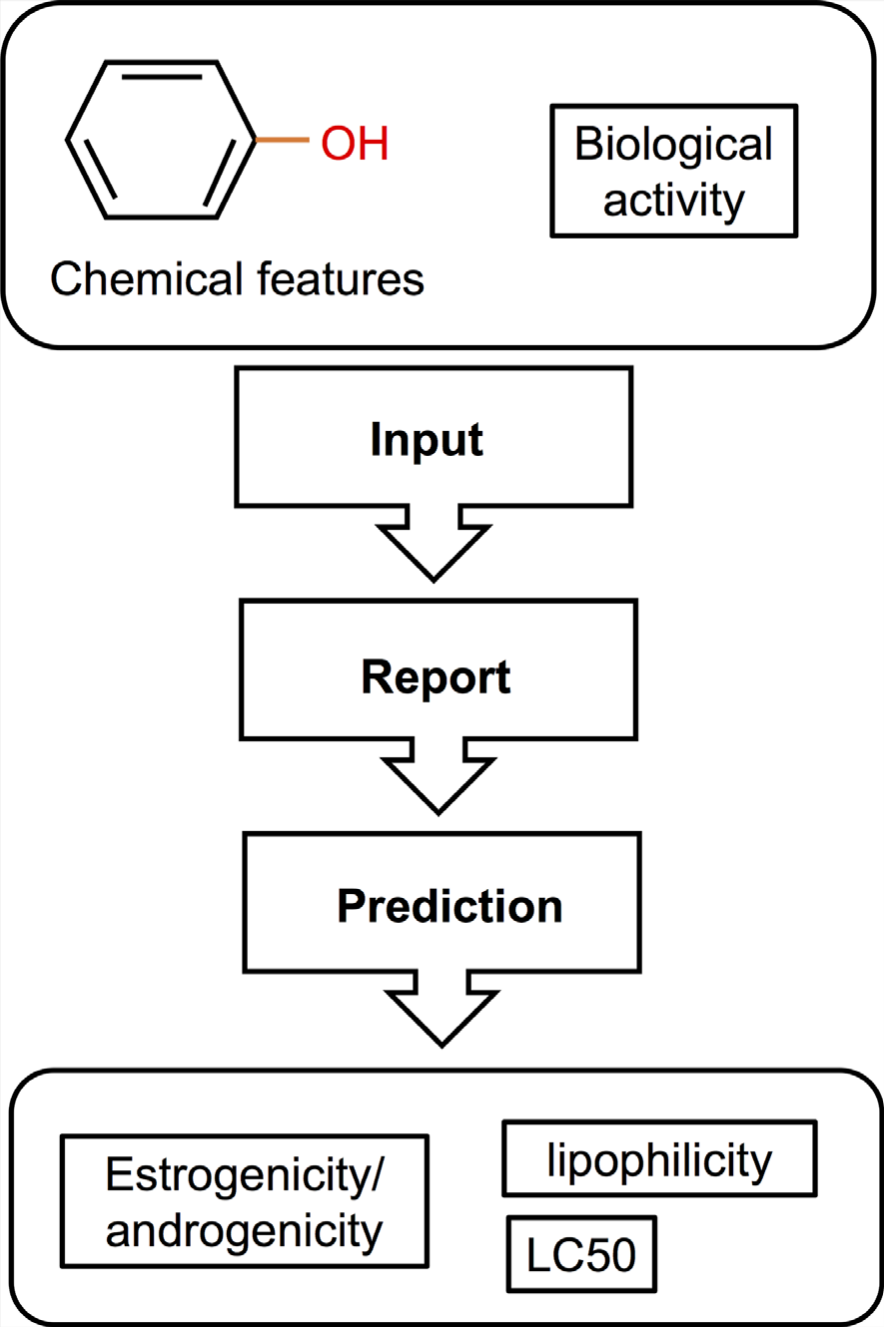
Schematic illustration of a QSAR method for screening of EDCs [adapted from Bohlen et al. (156), with author’s permission]. This type of *in silico* approach used a structure-activity relationship correlating the chemical features of compounds with their biological activity. The resulting data can then be used to predict the estrogenic/androgenic potential, toxicity, and lipophilicity of a compound or a complex mixture.

Computational methods used in drug development have been recently applied in EDCs research. These *in silico* approaches (illustrated in **Figure 4**) have the capacity to detect potential EDCs without the use of animals or cells, largely serving as an initial screening strategy that also attenuates the cost and time constraints of *in vivo* and *in vitro* approaches. So far, in fish, most computational models used structure–activity relationship methods which correlate the chemical structure of compounds with biological activity (**Figure 4** **)**. The biological activity is determined through a number of molecular descriptors such as molecular weight, hydrophobicity, topology, or electronic properties (220). Based on these descriptors, the estrogenicity, androgenicity, lipophilicity, and LC50 are then predicted (**Figure 4**). Comprehensive descriptions of various *in silico* methods suitable for EDC screening and research have been recently reviewed (220–222). In this section we discuss *in silico* approaches that have been used in EDC studies, including quantitative structure property relationship (QSAR), structural alerts or pharmacophore modeling, and molecular docking. QSAR prediction models have been developed to predict a particular activity or property of the molecule (e.g., molecular weight) of interest. Pharmacophore modeling detect features within the binding cavity that would ensure interactions between the biological target (receptor) and the potential ligands based on receptor structures (223–225) or aligns a set of known ligands in the absence of receptor structure to observe common features that may predict biological activity (226). Molecular docking, on the other hand, involves prediction of the ligand conformation and its position and orientation within binding sites and assessment of the binding affinity (227). This approach is utilized as a model for predicting the interaction between a small molecule and a protein at the atomic level, allowing for characterization of the behavior of molecules in the binding site of target proteins (228). Nevertheless, *in silico* methods have been only recently applied to predicting the effects of EDCs in fish and studies are scarce.

### QSAR/Structural Alerts

Potential EDCs in fish were screened using data from structural alerts and *in vitro* and *in vivo* toxicological assays (151). Structural alerts represent potential estrogenic and androgenic endocrine activities based on *in vitro* studies and are used for indicating potential estrogenic and androgenic endocrine disruptors in fish. The model generated was then applied to a database of commercial substances to search for chemicals with estrogenic or androgenic effects. The combination of these methods was found to have a potential to further assess candidate estrogenic and androgenic EDCs. In largemouth bass, transcriptome profiling data was coupled with computational analysis to model the effects of chemicals in the ovary. First, a dynamic model representing the development of healthy ovaries from unexposed fish was constructed, and the responses of the transcriptomes from fish collected from polluted areas were mapped to enable the identification of clusters of genes affected at different stages of ovarian development. Then, using a robust database that provides information on chemical interaction with genes, proteins, and diseases (Comparative Toxicogenomic Database), potential chemicals associated with specific molecular responses on the ovary were identified (155). Another study, applied the QSAR method utilizing chemical features and mode of action to predict acute toxicity effects of EDCs in fish. This study, among other outcomes, determined that estrogen receptor binding affinity is dependent on the relationship between lipophilicity (expressed as octanol-water partition coefficient, Kow) and LC50, and that both EE2 and NP strongly bind to the ER (156).

### Molecular Docking

A molecular docking approach is relevant for predicting the binding of EDCs to hormone receptors and binding proteins. It has been used to obtain insights on the molecular interactions of environmental xenoestrogens (229). In killifish, a molecular docking approach demonstrated that BPA interacted with residues in *foxl2* (162). Chen et al. (158) evaluated the estrogenic potentials of NP and BPA through the use of molecular docking analysis in conjunction with a reduced life expectancy model in adult zebrafish. The analysis revealed an interaction potential of both NP and BPA to ERα. Another study employing a 3-D structure-based computational method, showed that the ligand binding domains of medaka ERs are similar in architecture and have conserved amino acid residues that interact with E2; the authors also found that NP and BPA form hydrogen bonds with medaka ER1 (147).

These and other emerging computer-assisted technologies shall allow for prediction of endocrine disrupting activities of chemicals with increasingly greater power and specificity and complement the toolbox of approaches used for screening EDCs, *in vitro*.

## Conclusion

In this review, we provided an overview of studies employing different approaches to evaluate effects of select endocrine disrupting compounds. The results from these studies showed that common aquatic pollutants that are weakly estrogenic such as NP, can elicit long-term endocrine disrupting responses. Not only are EDCs long-lasting in contaminated environments due to their stable chemical structures, their effects can persist over the lifetime of an organism and in unexposed progenies of exposed parents.

*In vivo* approaches in various fish species are used to evaluate a wide range of consequences from exposure to EDCs including effects on body and organ weight, cell differentiation and growth, protein and gene expression and enzyme activities, among other endpoints. They tend to be, however, labor- and cost-intensive and unsuitable for large-scale screening. *In vivo* experiments can also provide more direct inferences to actual changes in biological activity but cannot, however, elucidate cellular mechanisms involved in EDCs exposure. By contrast, *in vitro* assays usually take into account a limited number of processes impacted by EDCs, but allow for assessment of the direct effects of chemicals on a specific tissue or cells with high screening throughput, though, testing a wide range of potential EDCs *in vitro* can be time consuming. Moreover, it is challenging to make direct inferences of biological activity at an organismal level from *in vitro* approaches. Last, *in silico* approaches offer an alternative strategy for rapid and robust screening while optimizing best practices for considering the use and care of animals in research, summarized by refinement, reduction and replacement (“3R’s”; 230). *In silico* approaches, however, still need further validation *in vivo*. Together, the results from various studies employing different approaches underscore the severity of EDC exposure in wild populations of fish. Alone or combined, *in silico*, *in vitro* and *in vivo* approaches shall continue to provide robust assessments of endocrine activity or adverse effects of EDCs on multiple physiological processes of aquatic organisms, including fish. Ultimately, collaborative efforts would enable the assessment of EDC’s effects by employing all three approaches to provide a more comprehensive understanding of the underlying processes involved in the exposure to EDCs or to evaluate potencies of wastewater effluents. The outcome of these comprehensive studies could in turn demonstrate the biological changes that impact the survival of aquatic organisms, thereby informing government agencies in making effective policy decisions.

## Author Contributions

FC-B and AS conceived the work. FC-B drafted the original manuscript and prepared the figures and table. FC-B, AS, and DL revised the manuscript. AS supervised the work. All authors contributed to the article and approved the submitted version.

## Funding

This work was funded in part by grants from the National Science Foundation (IOS-1755016), the National Oceanic and Atmospheric Administration (NA18OAR4170347) to AS and DL, the National Institute of Diabetes and Digestive and Kidney Diseases (1R21DK111775-01), and the National Institute of Food and Agriculture (Hatch no. HAW02051-H) to AS.

## Conflict of Interest

The authors declare that the research was conducted in the absence of any commercial or financial relationships that could be construed as a potential conflict of interest.
